# Seasonal Dynamics of Benthic Infauna Communities in *Zostera marina* Meadows: Effects of Plant Density Gradients

**DOI:** 10.3390/biology14020153

**Published:** 2025-02-03

**Authors:** Natalia Anna Gintowt, Halina Kendzierska, Urszula Janas

**Affiliations:** Faculty of Oceanography and Geography, University of Gdańsk, al. Marszałka Piłsudskiego 46, 81-378 Gdynia, Poland; halina.kendzierska@ug.edu.pl (H.K.); urszula.janas@ug.edu.pl (U.J.)

**Keywords:** seagrass, macrozoobenthos, bioturbation, bioirrigation, marine biodiversity, coastal zone, Baltic Sea

## Abstract

*Zostera marina* meadows play a key role in the Baltic Sea ecosystem. They are characterized by high primary production and provide feeding and reproduction grounds for organisms. These characteristics vary due to year-round environmental changes and may be due to the characteristics of the meadows themselves. Organisms inhabiting seagrass meadows are involved in the transformation of substances from terrestrial runoff, and, through bioturbation and bioirrigation, affect biogeochemical processes in the sediments. This study aimed to determine the structure of benthic communities inhabiting *Z. marina* meadows and their bioturbation (BP_C_) and bioirrigation (IP_C_) potential as affected by seagrass density and seasonal changes. This study shows a positive correlation between the density of *Z. marina* and the structure of macrozoobenthos, as well as the bioturbation and bioirrigation potential of the studied communities. The autumn season stimulated the density of macrofauna and recorded the highest values of their potential activities indices. The presence of *Z. marina* positively affects macrozoobenthic communities and their functioning regardless of seagrass density, indicating that seagrass meadows inhabited by macrofauna are key biotopes that can support biogeochemical processes in the coastal zone more effectively than bare sand.

## Simple Summary

Seagrass meadows, especially those formed by eelgrass *Zostera marina*, are very important biotopes, crucial to the healthy functioning of the Baltic coastal ecosystems. These meadows provide feeding and breeding areas for many marine organisms. Plants and animals that form these biotopes play a key role in transforming organic matter and nutrients in the marine ecosystem. Organisms dwelling in the sediments of seagrass meadows alter them through bioturbation (sediment mixing) and bioirrigation (water flow in the sediment). The objective of our study was to determine how the density of plants that form a meadow and the season of the year affect the species composition, density, and activity of these organisms. The results show that the presence of seagrass increases the number of species in the meadows and that their abundance boosts the activity of organisms. The season also plays an important role in shaping the functioning of the meadows, with the majority of organisms found in autumn; benthic communities have also been proven to have the highest potential for activity during this season compared to the rest of the year. Overall, the presence of seagrass helps maintain coastal ecosystems much more effectively than bare sand.

## 1. Introduction

Coastal zones represent one of the most important areas in marine ecosystems. They are characterized by specific physicochemical conditions that uniquely shape the diversity of habitats and the organisms living in them [1]. These zones are under very high water dynamics, and their environmental conditions are strongly affected by rivers and anthropogenic factors. All of these factors, combined with conditions such as the type of sediment in a given habitat, strongly affect the formation of unique and heterogeneous habitats in these zones [2]. Various types of unique habitats can be observed on the coast of the Baltic Sea [1,3,4,5]. These habitats—including the seagrass meadows of *Zostera marina* Linnaeus, 1753—are characterized by high variability in environmental conditions and perform different functions in the ecosystem [6,7].

Seagrasses play an important role as an engineering biotope; as meadows, they can modify the direction of ocean currents and stabilize sediments, thus preventing bottom erosion, and are involved in the circulation of elements and matter and the flow of energy [8,9]. In addition, they play a crucial role as stores of chemical substances, e.g., carbon. Even though they occupy less than 0.2% of the ocean’s surface, they store up to 10% of the carbon entering the oceans annually [10,11]. The high capacity of seagrasses to absorb carbon compounds from the environment can result in a local reduction in the level of water acidification [12]. However, high heterogeneity is observed among submerged meadows. This can include both the species composition of the macrophytes that make up the meadows and the prevailing conditions, namely the physicochemical parameters and the density of shoots. Each meadow is different and is characterized by unique environmental conditions. It has also been shown that even meadows in the same area can be distinguished by different functionality [13,14,15].

Organisms living in underwater meadows perform diverse activities and contribute to the proper functioning of these ecosystems [16]. Meadows are home to organisms of various sizes and taxonomic groups and representatives of all kinds of trophic guilds, from filter feeders and grazing organisms to predators [17,18]. Benthic organisms are essential to the cycling of chemical elements and nutrients, which they affect directly through activities such as feeding, respiration, and excretion, or indirectly by altering the sediment structure through activities such bioturbation and bioirrigation [6,19]. Both of these activities positively affect sediment conditions: water flowing through the burrows increases sediment oxygenation, and the burrows themselves create an increased surface area for colonization by microorganisms. In addition, intensive bioturbation and bioirrigation cause an exchange of substances between water and sediment and can stimulate the transformation of organic matter in the sediment [20,21,22,23,24,25]. Due to their important role in ecosystems, bioturbation and bioirrigation have been relatively extensively studied over the years. Various types of methods have been used for this purpose, including the estimation of bioturbation as its actual measurement. Bioturbation intensity is estimated by calculating Bioturbation (BP_C_) and Bioirrigation (IP_C_) Potential Indices, and is a relatively simple and widely used method for determining the functionality of given benthic species or communities [26,27,28,29,30].

Bioturbation and bioirrigation are important processes, but neither has been extensively studied for underwater meadows. One of the few studies conducted to date in these ecosystems involved *Zostera noltei* Hornemann, 1832 meadows in France, and showed an inhibitory effect from the meadows on the intensity of bioturbation activity of organisms [31]. The Baltic underwater meadows remain unexplored in terms of the intensity of bioturbation taking place there, but are fairly well studied in such aspects as the taxonomic composition of plants and animals that form and inhabit underwater meadows, the functioning of the meadows as trophic networks observed in them, and the biological traits of the macrofauna species living there. The studies were carried out on a single- or multi-seasonal basis [32,33,34,35,36].

The research presented here focuses on the effects of *Z. marina* density on macrofauna communities and their bioturbation and bioirrigation activities, both of which affect the functioning of coastal habitats, but are so far poorly researched in the Baltic Sea. The research was conducted on a seasonal basis to determine the effect of environmental conditions on the benthic communities of seagrass meadows and their activities.

## 2. Materials and Methods

### 2.1. Study Area and Sampling Design

**Figure 1:**
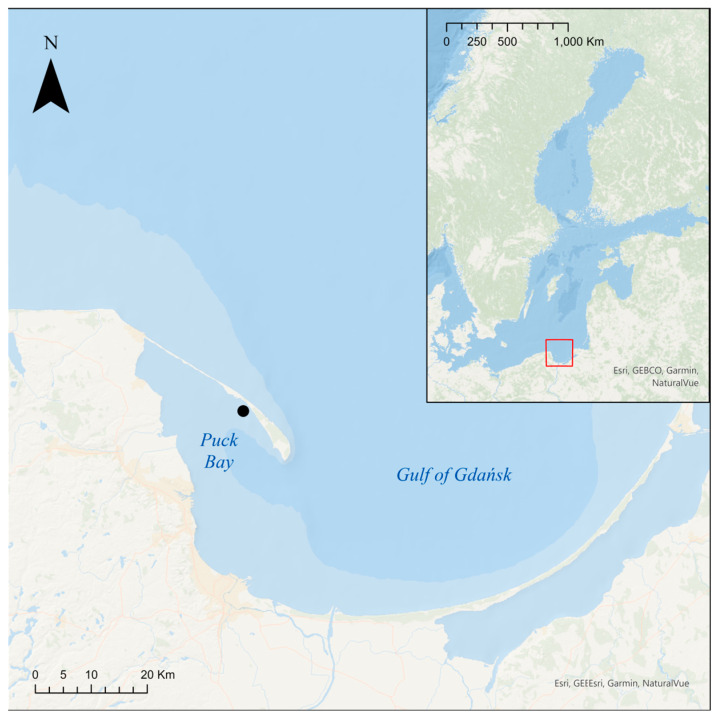
Study area with sampling site. The red rectangle indicates the location of the study area on a map of the Baltic Sea.

Macrozoobenthos samples were collected seasonally in November 2021 (Autumn) and February (Winter), May (Spring), and August (Summer) 2022 by divers at four density treatments in the *Z. marina* meadow (54°40.352′ N; 18°41.754′ E) on Długa Mielizna, a sandy shoal along the Hel Peninsula (Puck Bay) (Figure 1).

**Figure 2:**
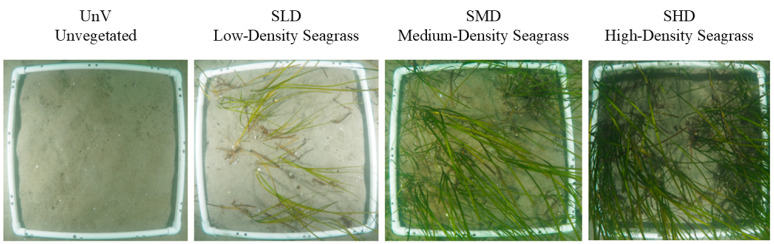
Photographs of the sampling sites with an increasing gradient of *Z. marina* shoots. The treatments were as follows: Unvegetated (UnV), Low-Density Seagrass (SLD), Medium-Density Seagrass (SMD), and High-Density Seagrass (SHD).

The unvegetated (UnV) site was located on bare sand about 2 m from a vegetated area (outside of the meadow) and three remaining sites—Low-Density Seagrass (SLD), Medium-Density Seagrass (SMD), and High-Density Seagrass (SHD)—were selected and delineated by a diver in the increased density gradient of *Z. marina* (Figure 2). Each replicate of macrofauna cores was located approx. 50–70 cm away from each other; meanwhile, sediment samples were subtracted approx. 30 cm from the fauna cores. Each *Zostera* treatment was taken from different *Z. marina* patches; the patches were about 4–6 m^2^ in size. A 50 × 50 cm frame was placed at each treatment site five times, and the number of *Zostera* shoots was counted. Bottom water temperature, salinity, and dissolved oxygen (DO) concentration were measured at each site. Five sediment cores (10 cm inner diameter, 25 cm of sediment) were collected at each site for macrofauna analysis.

### 2.2. Sediment Characteristics

Fifteen (three per macrofaunal treatment) additional intact sediment cores (3.5 cm inner diameter, 8 cm length) were collected to determine sediment parameters at each station. From five of these cores, the surface layer of the sediment (0–1 cm) was sliced and collected, and samples were subsequently frozen after being transported to the laboratory. The organic matter (OM) content was determined as the percentage of mass loss on ignition (LOI) (450 °C, 5 h) of the dried, homogenized sediment. The remaining two cores per macrofaunal treatment were used for grain size analysis. Samples were sieved using a shaker and a set of standard test sieves with mesh diameters of 2, 1, 0.5, 0.25, 0.125, and 0.063 mm [37]. Sediments were classified according to the Udden–Wentworth grain-size scale [38] based on the percentage of each class in the total sample mass.

### 2.3. Macrofauna

Animals were selected from the sediment, bypassing the sieving step that usually precedes the analysis of the macrofauna in a sample. The organisms were then sorted, and all taxa, except for Oligochaeta and *Marenzelleria* spp., were identified to the species level using specialistic identification guides [39,40,41]. The taxa were counted, and their wet mass was measured to determine their abundance and biomass per square meter. Shannon-Wiener’s (H’) diversity index and Pielou’s (J) evenness index were calculated using PRIMER 6 software (PRIMER-E Ltd., Ivybridge, UK).

### 2.4. Bioturbation Potential and Irrigation Potential

The wet mass of the organisms was used to calculate the Bioturbation Potential and Bioirrigation Potential indices. Despite the fact that many researchers use ash-free dry weight to calculate potentials, our previous studies of Baltic organisms showed that values of the indices calculated from wet weight and ash-free dry weight are in perfect agreement with each other r = 0.999 [26]. The Bioturbation Potential Index (BP_C_) at each site was the sum of the bioturbation potentials of individual taxa (BP_i_) [28,29] calculated according to the following equation:

(1) $${BP}_{c}=\sum {BP}_{i}\;where\;{BP}_{i}={\left(\frac{B_{i}}{A_{i}}\right)}^{0,5}*A_{i}*M_{i}*R_{i}$$

where B*_i_* is the biomass (wet mass g·m^−2^) and A*_i_* is the abundance (ind.·m^−2^) of taxon *i* in each sample, while mobility M*_i_* and sediment reworking R_i_ are categorical scores assigned to species *i* (Appendix A). In the scores assigned we focused on the macrofaunal impact on the sediment including epifauna living and influencing the sediment surface [26,28,29,42,43].

The Irrigation Potential Community Index (IP_C_) at each site was calculated by summing the irrigation potentials (IP_i_) calculated for each taxon [44]:

(2) $${IP}_{c}=\sum {IP}_{i}\;\mathrm{where}\;{IP}_{i}={\left(\frac{B_{i}}{A_{i}}\right)}^{0,75}*A_{i}*{BT}_{i}*{FT}_{i}*{ID}_{i}$$

where B_i_ is the biomass (g·m^−2^) and A_i_ is the abundance (ind.·m^−2^) for taxon *i* in each sample, while the feeding type FT_i_, burrow type BT_i_ and depth ID_i_ are scores for the trait categories assigned to each species.

Exponent 0.5 used in BP_C_ emphasizes the importance of organisms with high density and relatively low biomass, while exponent 0.75 used in IP_C_ emphasizes the activity of organisms with larger sizes but lower densities [30].

### 2.5. Data Analysis

A Principal Component Analysis (PCA) was conducted to determine the relationship between physicochemical conditions in sediments and bottom water and the variability between the sites. Data normality was tested using a Shapiro–Wilk test. A matrix with normalized data on bottom water temperature, surface sediment organic matter content, and the number of *Zostera* shoots was used in the analysis. The amount of <63 µm fraction and the biomass of *Z. marina* were not taken into account in this analysis due to the high correlation with organic matter content (r = 0.699) and the number of *Z. marina* shoots (r = 0.615), respectively. The abundance of macrofauna was square root transformed, and cluster analysis (Bray–Curtis similarity) was used to determine the similarity between macrofauna samples. Differences in total abundance, H’, J, BP_C_, and IP_C_ between the sampling sites and seasons were tested using PERmutational Multivariate Analysis of VAriances (PERMANOVAs) [45]. Data visualization and statistical analyses were performed in Microsoft Office 365 ProPlus, RStudio v4.30 (Venn diagram), and PRIMER v7 with PERMANOVA+ (PRIMER-E Ltd., Plymouth, UK).

## 3. Results

### 3.1. Environmental Conditions

Due to the close proximity of the sites, no differences in the parameters of bottom water were observed in a given season at the study sites. Variability in these parameters was observed only between seasons. The lowest temperature (3.9 °C) and the highest concentration of oxygen in the benthic water (13.97 mL/L) was observed in winter (Table 1). An inverse relationship was observed in summer when the temperature was highest (20.5 °C) and dissolved oxygen concentration was lowest (9.81 mL/L). Due to the shallow depth of the study sites (3 m), the water was well mixed and well oxygenated. Salinity at the surveyed sites was relatively stable, ranging from 6.8 in winter to 7.5 in autumn and summer. The organic matter content was relatively uniform among all sites during the studied seasons, and so was the sediment type, which was medium sand at all sites in all seasons. In all seasons, we were able to find seagrass patches at our designated density. The SLD treatment covered a density of 24–64 *Z. marina* shoots∙m^−2^ and the SHD treatment covered a density of 148–240 shoots∙m^−2^.

**Figure 3:**
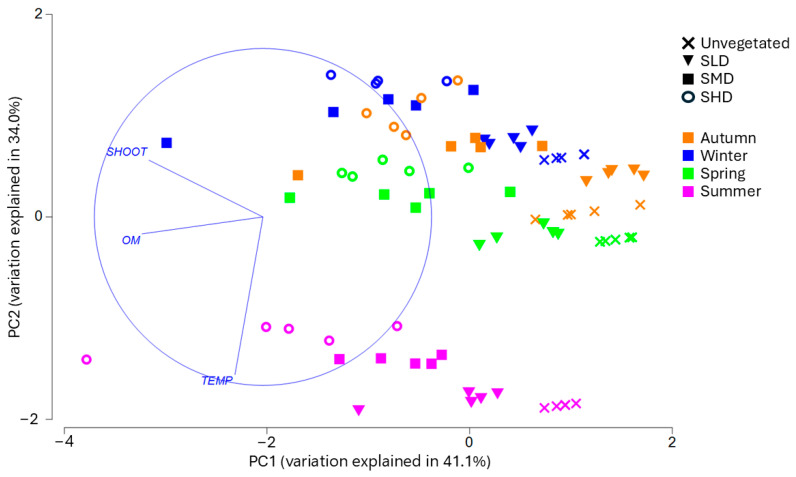
PCA results. Variables included in the PCA are bottom water temperature (TEMP), organic matter (OM) content of surface sediments and the number of *Zostera* shoots (SHOOT). Unvegetated (UnV), Low-Density Seagrass (SLD), Medium-Density Seagrass (SMD), and High-Density Seagrass (SHD).

The PC1 axis resulting from PCA explains 41.1% of the total variance (eigenvalue 1.23), with OM and the number of shoots being the most important explanatory factors (Table 2). PC1 and PC2 (eigenvalue 1.02) together explain 75.1% of the total variance (Figure 3). Temperature, with a coefficient of 0.936, contributed the most to the distribution along the PC2 axis.

### 3.2. Macrofauna

In the present study, we observed 29 taxa of benthic macrofauna in *Z. marina* meadows in Długa Mielizna in 2021–2022 (listed in Appendix Table A1). The species with the highest frequency of occurrence were the polychaete *Hediste diversicolor* (O.F. Müller, 1776) (99% of all collected cores) and the mud snails *Peringia ulvae* (Pennant, 1777) (97%) and *Ecrobia ventrosa* (Montagu, 1803) (85%). Other species present in more than 70% of the samples included Oligochaetes, polychaetes of the genus *Marenzelleria*, the bivalves *Cerastoderma glaucum* (Bruguière, 1789) and *Mya arenaria* Linnaeus, 1758, and the crustacean *Cyathura carinata* (Krøyer, 1847). In all of the studied seasons, the lowest species richness was noted at the unvegetated site. The greatest difference between the number of species at the surveyed sites was observed in summer, when only eight taxa were found at the site without vegetation, while 21 species were found at the SMD and SHD sites (Figure 3). The highest number of taxa at a single site was 23, observed in spring at the SMD site. At none of the bare sand treatments did the number of taxa exceed 10.

**Figure 4:**
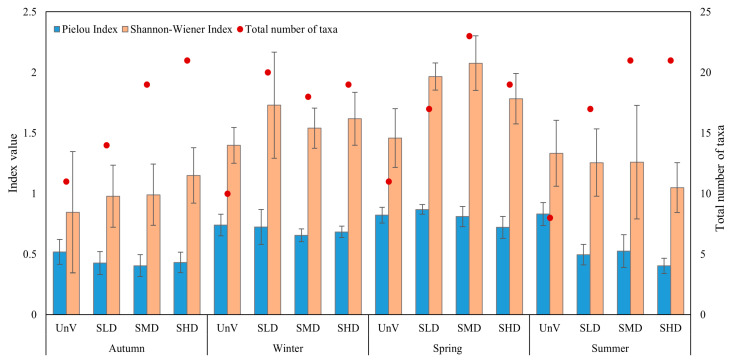
Macrofauna community descriptors (mean ± SD), Pielou index, Shannon–Wiener and total number of taxa diversity index by season following a spatial gradient of increasing shoot density: Unvegetated (UnV), Low-Density Seagrass (SLD), Medium-Density Seagrass (SMD), and High-Density Seagrass (SHD).

The highest H’ values were recorded at the sites covered with vegetation in spring (Figure 4). Statistically significant differences were found between the studied seasons (Table 3). There were no significant differences in the *Z. marina* gradient treatments. The Pielou index showed statistically significant differences between the sites and the seasons, as well as in their interaction—season x site. The autumn season was characterized by the lowest evenness values, which were due to the high dominance of *P. ulvae* in the macrofauna communities.

**Figure 5:**
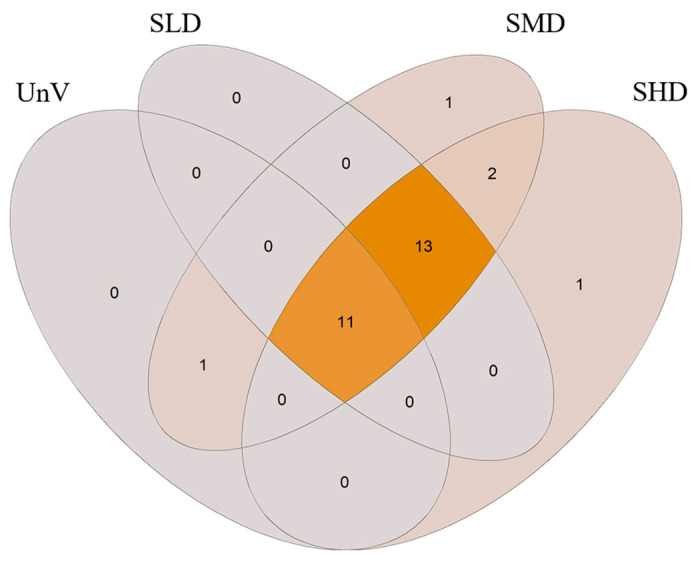
Venn diagram showing the number of taxa unique or common to different *Zostera* treatments—Unvegetated (UnV), Low-Density Seagrass (SLD), Medium-Density Seagrass (SMD), and High-Density Seagrass (SHD).

Analysis of the Venn diagram showed that 11 of the observed taxa were present in both the meadows and the bare sand area (Figure 5). Furthermore, 13 taxa were unique to *Z. marina* meadows. Four taxa were observed only in areas of dense seagrass: *Rhithropanopeus harrisii* (Gould, 1841), *Fabricia stellaris* (Müller, 1774), *Corophium volutator* (Pallas, 1766), and *Gammarus zaddachi* Sexton, 1912. The taxon found only on bare sand and at medium seagrass density was the amphipod *Bathyporeia pilosa* Lindström, 1855.

**Figure 6:**
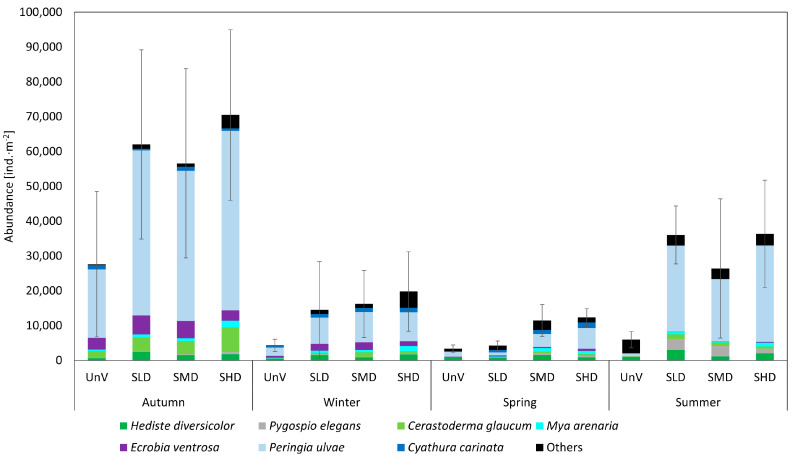
Abundance of macrofauna [ind.∙m^−2^] at the sampling sites in four seasons. Values are presented as means with standard deviation indicated. Unvegetated (UnV), Low-Density Seagrass (SLD), Medium-Density Seagrass (SMD), and High-Density Seagrass (SHD). ‘Others’ include 22 species with the lowest abundance (less than 5% of the total abundance).

Sites with the lowest biodiversity, i.e., those that were not overgrown with seagrass in any season, were also characterized by the lowest density of macrofauna. Our research indicates a small number of epifaunal species. At a few sites, less than 1% of the total macrofauna belonged to epifauna, but in most treatments, epifauna accounted for approx. 5% of all the fauna abundance. In terms of density, snails of the species *P. ulvae* dominated in all the study sites (12–76%; Figure 6). Mud snails *E. ventrosa* and clams *C. glaucum* contributed up to 14% and 10%, respectively, to the total abundance of organisms. We observed large differences between the abundance of organisms in all seasons (PERMANOVA, *p* < 0.01; Table 3). The highest abundance of organisms was observed in autumn, and large numbers of organisms were also observed in the meadows in summer. The fewest organisms were observed at all sites in spring. The difference between the abundance of macrofauna at the UnV and *Zostera* sites was statistically significant (PERMANOVA, *p* < 0.001; Table A3). In some months, lower densities of organisms were observed at the SMD sites than at the SLD and SHD (e.g., in autumn and summer).

### 3.3. Bioturbation and Bioirrigation

**Figure 7:**
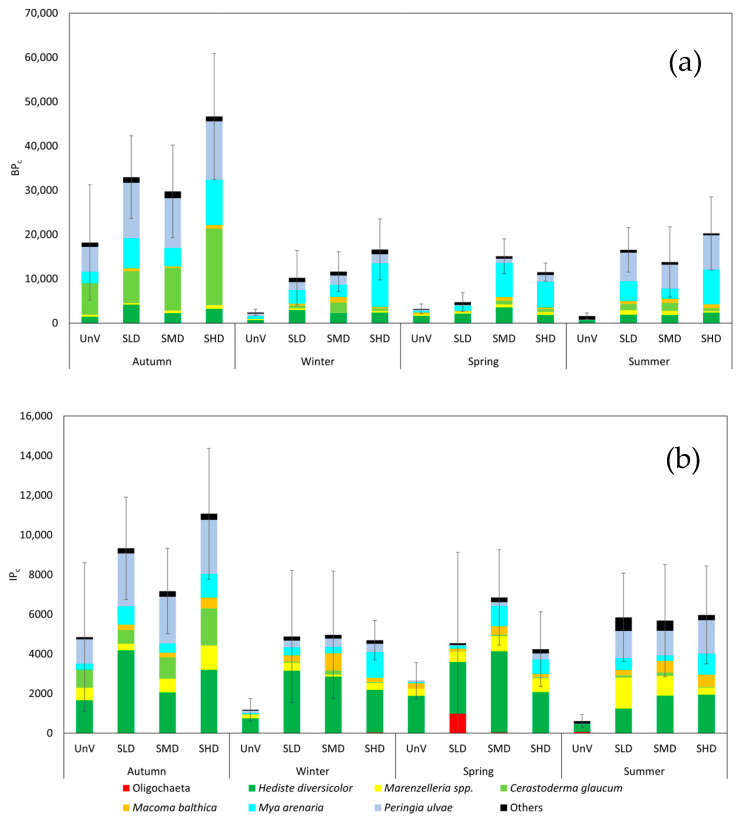
Functional indices (**a**) BP_C_ and (**b**) IP_C_ of the benthic community in cores collected from the sampling sites (n = 5). Values are presented as means with standard deviation indicated. Unvegetated (UnV), Low-Density Seagrass (SLD), Medium-Density Seagrass (SMD), and High-Density Seagrass (SHD). ‘Others’ include 22 species with the lowest abundance (less than 5% of the total abundance).

Both the bioturbation potential index and the bioirrigation potential index were higher at sites with higher seagrass density than at bare sand (Figure 7). In most seasons, the highest values of the bioturbation index were determined at the SHD site with the highest seagrass density. The bivalves *C. glaucum* and *M. arenaria* and *P. ulvae* snail were the main contributors to BP_C,_ followed by the fourth most abundant taxon, *H. diversicolor*. The lowest index was recorded at the sites not covered with seagrass, and was relatively similar (<5000) in all seasons except autumn; the difference between UnV and all *Zostera* treatments was statistically significant. Autumn was also the only season that was different from all of the other studied seasons (PERMANOVA, *p* < 0.001; Table A3). Other taxa than those responsible for BP_C_ were involved in the formation of bioirrigation potential. Polychaetes of the species *H. diversicolor* contributed the most to the formation of IP_C_, followed by the snails *P. ulvae*. *C. glaucum*, although also present, was not as important at most sites, except those surveyed in autumn. The highest values of the indices were observed in autumn, with the highest BP_C_ (35 162) and IP_C_ (11 071) being at the SHD site (Figure 7). As with BP_C_, the only season that differed statistically significantly from the other seasons was autumn, and only the UnV site differed from the other sites in terms of *Zostera* treatments (PERMANOVA, *p* < 0.001; Table A3).

## 4. Discussion

### 4.1. Zostera Meadows

The coastal zone is a dynamic environment characterized by very good mixing and oxygenation of water, resulting in a lower probability of water stratification [46]. The bottom water in the study area was well oxygenated and the salinity was uniform in all of the study sites. It is more likely that other parameters, such as sediment characteristics or temperature, had a greater impact on shaping the diversity of the benthic community. Research suggests that seagrass meadows are particularly attractive habitats for benthic organisms due to the accumulation of large amounts of organic matter in the sediments [11,47,48]. This is largely due to the filtering function of meadows in the coastal zone; they have the ability to trap and retain organic matter [49] and prevent sediment erosion [50]. Our results, however, did not confirm this relationship. A possible explanation for this is the fact that our research focused on seagrass patches rather than large seagrass meadows, which may accumulate less organic matter. In the past, Jankowska et al. [47] indicated that the organic matter content in the surface layer (up to 10 cm) of the sediments was not correlated with seagrass density in this area.

Our research has shown that *Zostera* meadows occur in the bay all year round, and in each season of the year, we are able to find fragments of meadows with a relatively high density of seagrass. Although the density of grass at each site varied from season to season in our study, we observed seagrass densities reaching up to at least 180 shoots∙m^−2^, even in the winter season, considered unfavorable for seagrass growth. Thus, the density of underwater meadows observed in our study appears to be relatively typical of Puck Bay. To the best of our knowledge, however, meadows of varying density and species composition of the macrophytes forming them are also observed in Puck Bay. Jankowska et al. [51] observed the highest density of seagrass in summer, and the density of *Z. marina* reached 200 shoots∙m^−2^, while in winter, the observed meadows were much sparser, and the number of shoots was only about 55 m^−2^. However, compared to other *Z. marina* sites in the Baltic Sea, it can be concluded that our meadows are characterized by relatively low densities. While conducting research in the Baltic Archipelago, Rodil et al. [35] observed that meadows there could reach a density of up to 800 shoots∙m^−2^, but those that were sheltered had lower densities of 150–250 shoots∙m^−2^.

### 4.2. Macrozoobenthos

The study of the macrofauna of underwater *Z. marina* meadows in Puck Bay presented here revealed the presence of 29 taxa at the surveyed sites, indicating the high taxonomic richness of the area. Many of these species were observed both in the underwater meadows and at the sites without vegetation, but the vegetated sites were always characterized by higher species diversity. This phenomenon underscores the importance of *Z. marina* meadows in shaping the coastal biodiversity, as described previously [3,32,35,52,53]. Thirteen of the observed taxa were specific to submerged meadows, regardless of their density. A similar pattern of the presence of specific species associated solely with vegetation was reported previously [16,33,54].

Interestingly, our study does not show a clear effect of meadow density on species composition; most species were simply present in vegetated areas, and only a few species were associated with specific levels of seagrass density: *R. harrisii* (SHD), *C. volutator* and *G. zaddachii* (SMD + SHD), and *B. pilosa* (UnV + SMD). Some researchers have shown a positive correlation between seagrass complexity and the diversity of macrofaunal communities [35,55]. In Puck Bay, *Z. marina* forms meadows together with other macrophyte species, such as *Potamogeton* spp., *Zannichelia palustris* L., *Stuckenia* spp., and *Ruppia* spp. [13,33,56]. We chose monospecific meadows for our study because we wanted sites to be homogeneous in terms of meadow density and species composition, even though mixed meadows may harbor a more diverse macrofaunal community [35,57].

Regarding the density of macrofauna, we found a typical pattern: higher abundance and biomass of organisms were recorded at vegetated sites compared to areas with bare sand. Overall, as indicated by other researchers [16,33,52,58], seagrass had a positive effect on the abundance of benthic taxa, but we did not observe a correlation between the density of macrofauna and the density of plants. Dense underwater meadows are not necessarily an indicator of diverse and densely populated macrofauna biotopes, and there are even studies showing a positive effect of habitat fragmentation on the density and species richness of macrofauna [59]. Rodil et al. [35], on the other hand, showed that the density of organisms and grasses is not a linear relationship.

Temperature was the second factor that explained most of the differences in macrozoobenthic communities and was closely related to the sampling season. Studies have shown large changes throughout the year in the number and diversity of species [51,58,60]. Some mobile macrofauna species are known to migrate to deeper parts of the coastal zone in search of more favorable environmental conditions during the year [61]. Ten of the species observed in submerged meadows occurred throughout the year; these were mainly the most abundant species with limited mobility, such as *C. glaucum* and *Macoma balthica* (Linnaeus, 1758). The highest number of organisms was found in autumn, which may be related to favorable environmental conditions for life and reproduction. Previous studies [51] have shown that the highest density and biomass of organisms in seagrass meadows were observed in the summer of 2011, so high biodiversity in the autumn season may indicate an extension or shift in favorable living conditions for organisms. These findings underscore the key role of temperature in shaping the diversity of macrozoobenthos communities.

The H’ index showed no differences between the treatments, but there were differences between seasons. Previous studies have shown differences in H’ between bare sand and meadow [33,58] and a relationship between H’ and the number of grass seedlings [62]. The lack of differences in the values of our indices may be due to the relatively homogeneous and similar environmental conditions prevailing at the treatments studied. However, in most cases, we observed larger differences in the J index at sandy sites than in the meadow. This is due to the fact that the meadows were often dominated by *P. ulvae*, a snail species that is typical and common in meadows.

### 4.3. Bioturbation and Bioirrigation

The occurrence of seagrass meadows affected both indices of organism activity, i.e., BP_C_ and IP_C_. In both cases, we observed higher values of the indices for the vegetated treatments than for those without vegetation, and there was also a strong seasonal variation between the values of the indices. The BP_C_ values recorded for the *Zostera* meadows are slightly lower in autumn and significantly lower in the other seasons compared to BP_C_ (calculated for wet mass) determined for the Vistula Plume [26]. This study was conducted on a similar type of sediment, but, due to the greater depth, a large number of intensely bioturbating taxa (*M. balthica*, *Marenzelleria* spp., *H. diversicolor,* and *M. arenaria*) dominated in the plum areas. Queirós et al. [63] showed that, in communities of muddy bottoms, BP_C_ was the highest in summer and autumn. High seasonal variation in the BP_C_ index was also observed in the western part of the Baltic Sea [64].

Our results indicate that autumn was a favorable season for the development of seagrass meadows and the activity of macrofauna due to favorable environmental conditions. As a result, we recorded high values of BP_C_ and IP_C_ indices, which reflect the potential for intense activity of organisms during this season.

The research presented here shows significant differences in the contribution of individual macrofaunal species to the formation of bioturbation and bioirrigation potential of benthic communities in the studied biotopes. The bioturbation potential is most strongly shaped by bivalves of the species *C. glaucum* and *M. arenaria*, which were the dominant species in the macrofauna biomass. Previous studies conducted in the Gulf of Gdańsk have shown a strong correlation between the BP_C_ index and the biomass of the observed organisms [26]. Our previous studies have also shown the dominance of bivalves in creating bioturbation potential. Although both indices, BP_C_ and IP_C_, are calculated based on the abundance and biomass of organisms, the coefficients used in the calculations to determine the aforementioned processes emphasize the contribution of specific species to the formation of the indices, and not necessarily those that dominated the abundance or biomass of organisms in the meadows. We observed such a phenomenon in the case of the index of bioirrigation potential, IP_C_. For this index, we observed a strong dominance of the polychaete *H. diversicolor* in its formation, as well as a relatively high proportion of mud snails *P. ulvae.* Numerous studies have shown that *H. diversicolor* is a very effective bioturbator and bioirrigator [65,66], even though, in our research, it did not significantly contribute to the abundance of organisms in the meadows. On the other hand, the high contribution of snails to the bioirrigation potential of organisms is more surprising. *P. ulvae* occurs relatively shallowly in the sediment and burrows only a few centimeters deep into the sediment without forming burrows. However, the high density of these snails indicates that this species can have a major impact on bioturbation and bioirrigation in sediments. The study by Andersen et al. [67] showed that the high density of *P. ulvae* causes a significant increase in the rate of sediment erosion. This was due to the intense movement of snails on the sediment surface and in the sediment, resulting in bioturbation and increased the permeability of the sediment to water. Thus, although *P. ulvae* does not create typical burrows, its presence in high densities can significantly affect bioturbation and bioirrigation.

BP_C_ and IP_C_ indices indicated both seasonal variation and variation related to *Zostera* density. Bioturbation and bioirrigation are very complex processes that are determined by numerous factors affecting the functioning of benthic organisms. In general, it can be said that both indices showed higher values in the meadows than in nearby unvegetated areas. Unfortunately, bioturbation or bioirrigation activity in seagrass meadows has not been studied much to date, neither by direct nor indirect methods. There are few studies focusing on bioturbation in *Z. marina* meadows. Bernard et al. [31] conducted direct experiments using luminescent markers to determine the exact bioturbation in seagrass meadows. However, they showed a correlation opposite to that presented in our study. Bernard et al. [31] showed that sediment particle mixing processes were less intense in meadows than in unvegetated areas, indicating a mitigating effect observed in submerged meadows. In summary, the results of our study indicate that bioturbation and bioirrigation in seagrass meadows are very complex and dynamic processes. They vary depending on local environmental conditions and the selected method of measurement or estimation. The different conclusions of our and Bernard’s research underscore the need for further and more comprehensive research in this area. Understanding the processes performed by organisms is crucial to assessing the role of seagrass meadows and seagrass itself in the functioning of benthic coastal ecosystems.

## 5. Conclusions

Our study highlights the key role of seagrasses in shaping the structure and functionality of benthic communities. Even meadows with relatively low densities have a positive impact on the biodiversity and functionality of the biotope. Both the overall density and species richness of organisms and potential for bioturbation BP_C_ and bioirrigation IP_C_ were significantly higher in the meadows than in the nearby sandy bottom without vegetation. Moreover, seasonal dynamics play a crucial role in shaping macrobenthic communities and their potential functioning. Autumn proved to be the most stimulating season, having the highest abundance of organisms and favoring increased BP_C_ and IP_C_. Our results underscore the ecological importance of seagrass meadows and the importance of these endangered habitats not only as reservoirs of biodiversity, but also as drivers of the ecological functioning of coastal biotopes.

## Figures and Tables

**Table 1:** Sediment characteristics and environmental parameters measured in bottom waters at the study sites in all seasons at the studied treatments—Unvegetated (UnV), Low-Density Seagrass (SLD), Medium-Density Seagrass (SMD), and High-Density Seagrass (SHD).

Season	Site	Temperature [℃]	Oxygen [mL/L]	Salinity	LOI [%] 0–1 cm	Number of *Zostera* shoots
Autumn	UnV	7.8	11.86	7.5	0.26	0
	SLD	7.8	11.86	7.5	0.20	48–64
	SMD	7.8	11.86	7.5	0.26	116–144
	SHD	7.8	11.86	7.5	0.24	164–240
Winter	UnV	3.9	13.97	7.3	0.27	0
	SLD	3.9	13.97	7.3	0.28	28–52
	SMD	3.9	13.97	7.3	0.32	112–132
	SHD	3.9	13.97	7.3	0.28	148–184
Spring	UnV	9.9	12.56	6.8	0.23	0
	SLD	9.9	12.56	6.8	0.27	24–44
	SMD	9.9	12.56	6.8	0.29	98–140
	SHD	9.9	12.56	6.8	0.27	148–180
Summer	UnV	20.5	9.81	7.5	0.25	0
	SLD	20.5	9.81	7.5	0.29	28–44
	SMD	20.5	9.81	7.5	0.28	96–124
	SHD	20.5	9.81	7.5	0.32	156–192

**Table 2:** Percentage of variation and coefficients in linear combinations of variables forming PCs.

Variable	PC1	PC2	PC3
Variation [%]	41.1	34.0	24.9
Bottom water temperature	−0.166	−0.936	−0.310
Organic matter content of surface sediments	−0.718	−0.101	0.689
Number of *Zostera* shoots	−0.676	0.337	−0.655

**Table 3:** Results of PERMANOVA analysis for differences in Abundance, BP_C_, IP_C_, Pielou Index, and Shannon–Wiener Index. Bold—statistically significant differences. df stands for deegrees of freedom.

		Abundance	Pielou Index	Shannon Index	BP_C_	IP_C_
Site	df	3	3	3	3	3
	MS	5838.3	1.0872 × 10^7^	1.2916 × 10^7^	12,109	1.9954 × 10^7^
	Pseudo-F	9.6171	3.0852	1.5442	24.513	4.0247
	p(perm)	**0.001**	**0.035**	0.184	**0.001**	**0.001**
Season	df	3	3	3	3	3
	MS	10,678	5.0784 × 10^7^	7.4832 × 10^7^	10,608	3.4079 × 10^7^
	Pseudo-F	17.589	14.411	8.947	21.475	6.8738
	p(perm)	**0.001**	**0.001**	**0.002**	**0.001**	**0.001**
Site x Season	df	3	9	9	9	9
	MS	1797.1	8.3333 × 10^6^	1.6627 × 10^7^	2137.2	5.7653 × 10^6^
	Pseudo-F	2.9603	2.3647	1.988	4.3267	1.1629
	p(perm)	**0.001**	**0.025**	0.057	**0.001**	0.239

## Data Availability

Data are contained within the article.

## Appendix A

**Table A1:** Categorical scores assigned to each taxon for BP_C_ and IP_C_ indices were calculated according to Solan et al. [28], Villnäs et al. [29], Queirós et al. [42], Miernik et al. [26], and Wrede et al. [43] (modified). Modifications to scores were made to focus on the impact of macrofauna on the sediment, including epifauna living and influencing the sediment surface. Mobility (M_i_) scores: 1—movement on the sediment surface, sessile; 2—limited movement; 3—slow, free movement through sediment; 4—free movement through sediment. Reworking types (R_i_): 1—epifauna; 2—surficial modifiers; 3—upward or downward conveyors; 4—biodiffusors. Burrow types (BT_i_): 1—epifauna or internal irrigation (i.e., siphons); 2—open irrigation (i.e., Y- or U-shaped burrow); 3—blind ended burrow. Feeding types (FT_i_): 1—surface filter feeders; 2—predators; 3—deposit feeders; 4—subsurface filter feeders. Irrigation depth (ID_i_): 1—0–1 cm; 2—1–3 cm; 3—3–6 cm; 4—6–10 cm; 5—10–15 cm.

Taxa	BP_C_		IP_C_		
	M_i_	R_i_	Bt_i_	Ft_i_	ID_i_
Oligochaeta	3	2	3	3	4
*Fabricia stellaris* (Müller, 1774)	2	1	3	1	2
*Hediste diversicolor* (O.F. Müller, 1776)	4	3	2	3	5
*Marenzelleria* spp.	4	4	3	3	5
*Pygospio elegans* Claparède, 1863	2	2	3	3	3
*Streblospio shrubsolii* (Buchanan, 1890)	2	2	3	3	2
*Cerastoderma glaucum* (Bruguière, 1789)	3	2	1	1	1
*Macoma balthica* (Linnaeus, 1758)	3	4	1	3	4
*Mytilus trossulus* Gould, 1850	1	1	1	1	1
*Mya arenaria* Linnaeus, 1758	3	4	1	1	2
*Ecrobia ventrosa* (Montagu, 1803)	2	3	1	3	1
*Peringia ulvae* (Pennant, 1777)	2	3	1	3	2
*Theodoxus fluviatilis* (Linnaeus, 1758)	1	1	1	3	1
*Amphibalanus improvisus* (Darwin, 1854)	1	1	1	1	1
*Bathyporeia pilosa* Lindström, 1855	4	4	3	3	2
Chironomidae	2	2	1	1	1
*Corophium multisetosum* Stock, 1952	2	2	2	3	3
*Corophium volutator* (Pallas, 1766)	2	2	2	3	3
*Crangon crangon* (Linnaeus, 1758)	4	2	1	3	1
*Cyathura carinata* (Krøyer, 1847)	2	2	1	3	1
*Gammarus oceanicus* Segerstråle, 1947	1	1	1	3	1
*Gammarus salinus* Spooner, 1947	1	1	1	3	1
*Gammarus zaddachi* Sexton, 1912	1	1	1	3	1
*Idotea balthica* (Pallas, 1772)	1	1	1	3	1
*Idotea chelipes* (Pallas, 1766)	1	1	1	3	1
*Idotea granulosa* Rathke, 1843	1	1	1	3	1
Insecta larvae	1	1	1	3	1
*Lekanesphaera hookeri* (Leach, 1814)	1	1	1	3	1
*Rhithropanopeus harrisii* (Gould, 1841)	4	2	1	2	1

**Table A2:** PERMANOVA pairwise results for differences in IP_C_ and Shannon–Wiener Index. Unvegetated (UnV), Low-Density Seagrass (SLD), Medium-Density Seagrass (SMD), and High-Density Seagrass (SHD). Bold—statistically significant differences.

	Pair	IP_C_		Shannon–Wiener	
		t	*p*	t	*p*
**Site**	**UnV, SLD**	2.721	**0.002**		
	**UnV, SMD**	3.202	**0.002**		
	**UnV, SHD**	3.202	**0.001**		
	**SLD, SMD**	0.521	0.916		
	**SLD, SHD**	1.046	0.326		
	**SMD, SHD**	1.042	0.366		
**Season**	**Autumn, Summer**	2.881	**0.001**	0.162	0.858
	**Autumn, Winter**	3.175	**0.001**	4.308	**0.003**
	**Autumn, Spring**	3.105	**0.001**	4.308	**0.002**
	**Summer, Winter**	2.020	**0.014**	3.194	**0.009**
	**Summer, Spring**	2.432	**0.004**	3.194	**0.005**
	**Winter, Spring**	0.960	0.338	0.584	0.781

**Table A3:** PERMANOVA pairwise results for differences in the Abundance, Pielou Index, and BP_C_. Unvegetated (UnV), Low-Density Seagrass (SLD), Medium-Density Seagrass (SMD), and High-Density Seagrass (SHD). Bold—statistically significant differences.

		Abundance		Pielou Index		BP_C_	
	Pair	t	*p*	t	*p*	t	*p*
**UnV**	**Autumn, Summer**	2.891	**0.012**	3.624	**0.011**	3.224	**0.005**
	**Autumn, Winter**	1.912	**0.008**	2.982	**0.013**	3.089	**0.012**
	**Autumn, Spring**	2.187	**0.008**	3.644	**0.008**	2.886	**0.005**
	**Summer, Winter**	4.229	**0.008**	1.572	0.132	1.753	0.081
	**Summer, Spring**	2.973	**0.013**	0.190	0.858	2.699	0.051
	**Winter, Spring**	1.631	**0.008**	1.653	0.146	1.162	0.29
**SLD**	**Autumn, Summer**	2.774	**0.008**	1.384	0.216	3.625	**0.013**
	**Autumn, Winter**	2.630	**0.02**	3.284	0.016	3.383	**0.008**
	**Autumn, Spring**	4.287	**0.012**	1.941	0.093	6.757	**0.009**
	**Summer, Winter**	2.643	**0.009**	3.054	0.036	1.658	0.09
	**Summer, Spring**	4.038	**0.008**	1.338	0.256	4.735	**0.01**
	**Winter, Spring**	1.997	**0.006**	0.068	0.985	1.738	0.101
**SMD**	**Autumn, Summer**	2.350	**0.008**	1.653	0.145	2.076	**0.033**
	**Autumn, Winter**	1.924	**0.01**	0.071	0.903	3.317	**0.023**
	**Autumn, Spring**	2.966	**0.008**	7.332	**0.01**	2.973	**0.023**
	**Summer, Winter**	1.759	**0.015**	0.706	0.474	0.525	0.701
	**Summer, Spring**	1.923	**0.016**	4.000	**0.009**	0.877	0.457
	**Winter, Spring**	1.512	**0.025**	2.659	**0.014**	1.302	0.207
**SHD**	**Autumn, Summer**	2.864	**0.005**	1.136	0.325	3.517	**0.012**
	**Autumn, Winter**	2.874	**0.008**	1.033	0.351	4.008	**0.009**
	**Autumn, Spring**	3.702	**0.005**	5.252	**0.008**	7.453	**0.01**
	**Summer, Winter**	2.266	**0.009**	1.578	0.159	0.714	0.526
	**Summer, Spring**	2.713	**0.006**	4.420	**0.011**	2.493	**0.021**
	**Winter, Spring**	1.048	0.394	1.151	0.297	1.467	0.192
**Autumn**	**UnV, SLD**	1.186	0.061	0.185	0.883	1.193	0.138
	**UnV, SMD**	1.085	0.168	0.537	0.565	1.004	0.337
	**UnV, SHD**	1.507	**0.009**	0.731	0.454	1.585	**0.013**
	**SLD, SMD**	0.743	0.746	0.450	0.739	0.580	0.677
	**SLD, SHD**	1.719	**0.039**	0.719	0.634	1.833	0.113
	**SMD, SHD**	1.776	**0.013**	0.465	0.718	1.991	0.067
**Winter**	**UnV, SLD**	2.044	**0.002**	0.202	0.845	3.257	**0.011**
	**UnV, SMD**	2.352	**0.012**	2.177	**0.043**	5.223	**0.009**
	**UnV, SHD**	2.496	**0.013**	1.292	0.18	5.893	**0.008**
	**SLD, SMD**	0.868	0.601	1.964	**0.084**	0.644	0.726
	**SLD, SHD**	1.011	0.357	1.100	0.329	1.398	0.208
	**SMD, SHD**	0.891	0.583	0.794	0.302	1.191	0.274
**Spring**	**UnV, SLD**	2.024	**0.006**	0.674	0.883	1.425	0.164
	**UnV, SMD**	2.582	**0.009**	0.232	0.842	6.835	**0.011**
	**UnV, SHD**	3.046	**0.009**	1.998	0.094	6.585	**0.008**
	**SLD, SMD**	1.930	**0.019**	0.599	0.893	4.774	**0.005**
	**SLD, SHD**	2.433	**0.005**	0.051	0.992	4.194	**0.006**
	**SMD, SHD**	0.946	0.595	1.611	0.149	1.776	0.106
**Summer**	**UnV, SLD**	5.220	**0.01**	5.880	**0.015**	6.982	**0.013**
	**UnV, SMD**	3.559	**0.007**	4.136	**0.024**	3.971	**0.008**
	**UnV, SHD**	4.581	**0.017**	5.608	**0.008**	6.453	**0.011**
	**SLD, SMD**	1.135	0.279	0.412	0.667	0.959	0.403
	**SLD, SHD**	1.408	0.068	1.884	0.064	0.756	0.478
	**SMD, SHD**	1.184	0.242	1.955	0.081	1.196	0.263
